# 

**Published:** 1894-06

**Authors:** 


### CONTENTS—JUNE, 1894.—VOL. IX., NO. 12.

ORIGINAL COMMUNICATIONS—

Report of Parasitic Entozoa Encountered in General Practice in Texas During Over Forty Years. F. Herff, M. D., San Antonio . . 613

Syphilitic Epididymitis. Geo. W. Davis, M. D., Kansas City, Mo . . 616

Gastro-Hysteropexy as a Safe and Reliable Means of Correcting Prolapsus and Retro-Displacement of the Uterus. Young H. Bond, M. D., St. Louis, Mo . . . . . . . . . . . 618

Antipyretics. Dr. W. A. Archer, Houston. . . . . . . . . . . 627

Hydrosalpinx Prolapsed into Douglas' Pouch—Hydrosalpinx Drained Through the Uterus—Chronic Salpingitis and Ovaritis. Augustine H. Goelet, New York . . . . . . . . . . 630

ABSTRACTS OF CURRENT MEDICAL LITERATURE—

Department of Therapeutics. Edited by Prof. D. Cerna, M. D., Galveston . . . . . . . . . . 634

Department of Practice of Medicine. Edited by Prof. A. J. Smith, M. D., Galveston. . . . . . . . . . 639

CORRESPONDENCE—

Pot-Pourri. Dr. T. J. Pugh . . . . . . . . . . 640

SOCIETY NOTES—

Austin District Medical Society . . . . . . . . . . 641

Central Texas Medical Association, etc . . . . . . . . . . 641

EDITORIAL—

The Deadly Doctor . . . . . . . . . . 643

MEDICAL NEWS AND MISCELLANY—

Many Interesting Paragraphs . . . . . . . . . . 644

BOOK NOTICES— . . . . . . . . . . 647

PUBLISHERS' NOTES . . . . . . . . . . 655

